# An Acebuche Oil-Enriched Diet Prevents Early-Stage Cerebrovascular Alterations in the 5xFAD Mouse Model of Alzheimer’s Disease

**DOI:** 10.3390/nu18010172

**Published:** 2026-01-05

**Authors:** Lorenzo Guidotti, Dominga Lapi, Martina Lucchesi, Silvia Valori, Francesca Corsi, Lucia Giambastiani, Andrea Vornoli, Claudia Gargini, Maurizio Cammalleri, Massimo Dal Monte

**Affiliations:** 1 Department of Biology, University of Pisa, 56127 Pisa, Italy; l.guidotti1@student.unisi.it (L.G.); dominga.lapi@unipi.it (D.L.); martina.lucchesi@biologia.unipi.it (M.L.); s.valori1@studenti.unipi.it (S.V.); maurizio.cammalleri@unipi.it (M.C.); 2 Department of Pharmacy, University of Pisa, 56126 Pisa, Italymaria.gargini@unipi.it (C.G.); 3 Institute of Agricultural Biology and Biotechnology (IBBA), National Research Council, 56124 Pisa, Italy; lucia.giambastiani@ibba.cnr.it (L.G.); andrea.vornoli@ibba.cnr.it (A.V.); 4 Interdepartmental Research Center Nutrafood “Nutraceuticals and Food for Health”, University of Pisa, 56124 Pisa, Italy

**Keywords:** beta amyloid, cerebral microcirculation, neuroinflammation, blood–brain barrier breakdown, healthy diet, metabolic parameters

## Abstract

**Background/Objectives**: Alzheimer’s disease (AD) is a neurodegenerative disorder in which altered microvascular circulation participates in the pathogenesis. The lack of therapeutic treatments for AD makes the development of strategies aimed at preventing or delaying the disease onset urgent. In recent years, several studies have highlighted that a diet rich in antioxidants and anti-inflammatory compounds may positively impact AD development. In this study, we assessed the impact of a diet enriched with Acebuche (ACE) oil, an extra-virgin olive oil particularly rich in antioxidants and anti-inflammatory compounds, on AD progression in the 5xFAD mouse model. **Methods:** After weaning, wild-type (WT) and 5xFAD mice received the standard or the ACE oil-enriched diet. At 2, 4 and 6 months, the effects of the diet were evaluated on AD-related microvascular aberrancies, beta-amyloid (Aβ) formation, hypoxic state, blood–brain barrier (BBB) alterations, neuroinflammation and cognitive impairment. Metabolic parameters were also evaluated. **Results:** In 5xFAD mice, the ACE oil-enriched diet prevented alterations in cerebral microcirculation. Moreover, Aβ accumulation, downregulation of Aβ-degrading enzymes, hypoxia, BBB breakdown, neuroinflammation, and cognitive deficits were delayed by the ACE oil-enriched diet. However, some of these effects were reduced at 6 months, in concomitance with systemic metabolic changes, such as hepatic steatosis, evidenced in both WT and 5xFAD mice receiving the ACE oil-enriched diet. **Conclusions:** Overall, the present results represent proof of concept for the validity of early dietary interventions in AD prevention.

## 1. Introduction

Alzheimer’s disease (AD) is a widespread neurodegenerative disorder that progressively impairs cognitive and behavioral functions [1]. AD is characterized by deregulated processing and clearance of beta-amyloid (Aβ) and hyperphosphorylation of Tau protein, which lead to the formation of Aβ plaques and neurofibrillary tangles, respectively [2,3]. The dramatic rise in AD cases and the lack of effective treatments urge the search for novel strategies aimed at preventing or delaying AD onset [4].

Over the last few decades, several studies have progressively highlighted that systemic vascular pathologies such as hypertension and diabetes are associated with an increased risk of developing AD [5,6,7,8,9]. Furthermore, in AD patients, neuroimaging investigations have revealed the presence of cerebrovascular lesions, including infarcts, white matter lesions, and cerebral microbleeds, which contribute to cognitive decline [7,10,11,12]. This suggests that vascular factors affecting the cerebral microvasculature are likely to play an important role in AD pathogenesis. Interestingly, hypoperfusion consequent to altered cerebral vascularization leads to hypoxia, which in AD results in increased levels of Aβ by stimulating its production and inhibiting its degradation [13].

Intriguingly, Mediterranean Diet supplementation, which has been demonstrated to counteract the development and progression of hypertension and cardiovascular diseases [14,15,16], may play a positive role in reducing the impact of AD. Indeed, subjects with high adherence to this diet show about a 40% lower risk of developing AD [17,18], slower cognitive decline, reduced disease progression from mild cognitive impairment to AD, and improved cognition [19]. Additionally, in AD patients, high adherence to the Mediterranean diet is associated with a reduction in cerebral Aβ plaque deposits [20].

It is well-known that the main ingredient of the Mediterranean diet is extra-virgin olive oil (EVOO). Indeed, EVOO is the principal source of dietary fat, providing more calories than any other individual food, and it is likely to be responsible for a substantial part of the diet’s healthy effects [21,22]. Consistently, feeding aging rodents EVOO improves brain biochemical parameters, memory, and motor coordination, and reduces oxidative stress [23,24]. In different mouse models of AD an EVOO-enriched diet has been demonstrated to reduce Aβ deposition and Tau protein levels, and to improve memory performance [25,26,27]. In humans, the consumption of EVOO, either in the context of a Mediterranean diet or not, has been associated with an improvement in cognitive functions [28,29,30,31]. In addition, in subjects with mild cognitive impairment, daily consumption of EVOO for 6 months resulted in a reduction in BBB permeability [28].

Among EVOOs, the olive oil named Acebuche (ACE), coming from a wild olive tree (Olea europaea var. sylvestris) growing particularly in Andalusia in Southern Spain, shows twice the content of triterpene acids (including oleanolic acid and maslinic acid), higher levels of tocopherols, sterols, and a higher ratio of secoiridoids/ortodiphenols in comparison to other EVOOs [32]. It has been recently demonstrated that an ACE oil-enriched diet efficiently counteracts the increase in systemic blood pressure in a murine model of hypertension, yielding better results compared to a different EVOO-enriched diet [32,33]. Moreover, in a rat model of systemic hypertension, an ACE oil-enriched diet lowered blood pressure, counteracted aorta alterations in terms of morphology and responsiveness to vasoactive mediators, and decreased fibrotic and oxidative stress processes, thus showing important beneficial effects at the vascular level [34]. Additionally, in glaucoma models, the ACE oil-enriched diet exerted a neuroprotective action against the progression of neurodegeneration [35,36]. Accordingly, we investigated whether ACE oil might exert protective effects on the development and progression of AD and associated cerebral microvascular dysfunction, employing the 5xFAD mouse model, one of the most widely used and accessible transgenic models of AD. The 5xFAD mice overexpress five familial AD mutations, three on the human *APP* gene (Florida I716V, Swedish K670/M671L, and London V717I) and two on the human presenilin 1 (*PSEN1*) gene (L286V and M146L). Each mutation is driven by the mouse thymus cell antigen 1 promoter, which directs expression to forebrain neurons [37,38,39]. Due to these mutations, the 5xFAD murine model develops early cerebral Aβ deposition associated with gliosis, neuroinflammation, synaptic dysfunction, BBB impairment, and cerebrovascular alterations leading to neuronal loss and subsequent cognitive decline [39,40,41,42]. This murine model has been previously used by different research groups to demonstrate the beneficial effects of EVOO and its phenolic compounds in the prevention and treatment of AD [43].

## 2. Materials and Methods

### 2.1. Animals

Male 5xFAD mice (B6.Cg-Tg(APPSwFlLon, PSEN1*M146L*L286V)6799Vas/Mmjax) were purchased from Jackson Labs (Bar Harbor, Mainer, ME, USA) and bred in our laboratory with female C57BL/6J mice (Jackson Labs). Littermates were genotyped for *APP* and *PSEN1* transgenes through PCR with specific primers (Table 1). Mice that did not show the expression of the transgene were used as wild-type (WT) controls. In the present study, we used 2-, 4-, and 6-month-old mice. The number of mice used in the present study, as well as their suffering, was limited according to the 3Rs principles for the ethical usage of animals in scientific research. A total of 82 mice (41 WT and 41 5xFAD) were used. Animals were maintained under standard laboratory conditions with a 12 h light/dark cycle at 23 ± 1 °C, with food and water provided ad libitum.

### 2.2. Dietary Supplementation

Both WT and 5xFAD mice received the standard or the ACE oil-enriched diet immediately after weaning until 2, 4, and 6 months of age. To prepare the ACE oil-enriched diet, the standard diet, a commercial rodent chow (Teklad 2018, Envigo, San Giorgio al Natisone, Italy), was crushed into powder form and then homogeneously mixed with ACE oil (Aceite Acebuche Mudéjar, Monda, Spain) to reach a final concentration of 12% (*w*/*w*), following previous literature [32,33,35,36]. The mixture was used to produce new pellets that were maintained at 4 °C in the dark until daily use. The caloric density of the standard diet is 3.1 kcal/g, while that of the ACE oil-enriched diet is about 4.1 kcal/g. The standard diet contains 18.4% proteins, 6% fats and 44.2% available carbohydrates, while the ACE oil-enriched diet contains 16.2% proteins, 17.2% fats and 38.9% available carbohydrates.

### 2.3. Surgical Animal Preparation

WT and 5xFAD mice at 2, 4, and 6 months of age, receiving the standard or the ACE oil-enriched diet were anesthetized by intraperitoneal injection of Avertin (1.25% solution, 0.02 mL/g body weight), tracheotomized, and mechanically ventilated with room air and supplemental oxygen after induction of paralysis with tubocurarine chloride (1 mg/kg, intravenous). The ventilator settings were adjusted to keep blood gas levels within the physiological range, with continuous monitoring of end-tidal CO_2_. Periodic arterial blood samples (100 µL) were collected through a catheter inserted into the femoral artery for blood gas analysis. Body temperature was measured and maintained at 37.0 ± 0.5 °C using a heating stereotaxic frame [44]. The lateral tail vein, located on either side of the tail, was used for intravenous injection of the fluorescent tracer [fluorescein isothiocyanate (FITC) bound to dextran, molecular weight 70 kDa, 50 mg/100 g body weight, as a 5% wt/vol solution in 3 min] through a small-gauge needle (e.g., 27–30 G). Successively, to visualize the pial microvasculature, a closed cranial window (3 mm × 4 mm) was implanted above the left parietal cortex (stereotactic coordinates: posterior 2 mm to bregma; lateral, 3 mm to midline) [45]. During the drilling of the cerebral cortex, a cold saline solution was superfused on the skull to avoid overheating. The skull and the dura mater were removed, and the brain parenchyma was continuously superfused with artificial cerebrospinal fluid [46].

### 2.4. Pial Microcirculation Observation

A fluorescence microscopic technique was utilized in vivo to observe the pial microcirculation. The microscope (Orthoplan, Leitz, Wetzlar, Germany) was equipped with long-distance objectives [5×, numerical aperture (NA) 0.08; 10×, NA 0.20; 32×, NA 0.40], a 10× eyepiece, and a filter block (Ploemopak, Leitz) used for FITC and Thioflavin S detection. A 100 W mercury lamp provided epi-illumination and a heat filter prevented the overheating of the preparations (KG1, Leitz). The pial microvascular networks were visualized by a low-light-level camera (CCD-300, DAGE-MTI, Michigan City, IN, USA), and the real-time recordings were stored through a computer-based frame grabber (Pinnacle DC10 plus, Avid Technology, Burlington, MA, USA). For each microvascular network, vessel diameter and length were measured using a frame-by-frame computerized method (MIP Image, Institute of Clinical Physiology, National Research Council, Pisa, Italy), and pial arterioles were classified according to Strahler’s scheme [47,48,49]. Briefly, the terminal arterioles (order 1), or those that give rise to the capillaries (order 0), were identified first. The higher-order arterioles were then identified. For each vessel, the diameter was expressed as the mean and standard error of the mean of three diameter measurements taken in areas adjacent to the vessel itself, and the length was measured with successive repeated measurements along the entire length of the vessel between one branch and the next. By plotting the lengths and diameters obtained from the monitor images in stop-frame conditions, it was possible to construct a map of each microvascular network studied. In each animal, the number and order of vessels in a window of 400 μm × 400 μm were evaluated. The increase in microvascular permeability, an indicator of compromised BBB integrity, was quantified by evaluating the fluorescent dextran leakage from blood vessels and expressed as normalized gray levels (NGLs): NGL = (I − Ib)/Ib, where Ib was the baseline gray level at the microvasculature filling with fluorescence, and I was the value after 30 min of observation. The reported NGLs were obtained by averaging the measures acquired in five windows of 50 µm × 50 µm located outside the vessels. To identify the same regions of interest, a computer-assisted device for XY movement of the microscope table was used. The density of functional capillaries, a key indicator of tissue blood supply, was measured by a computerized method (MIP Image, Institute of Clinical Physiology, National Research Council) in an area of 150 μm^2^. Aβ extracellular deposition was evaluated by Thioflavin-S (ThS) fluorescence. The pial layer was superfused with artificial cerebrospinal fluid containing 250 mM ThS at 37.0 ± 0.5 °C. ThS fluorescence intensity was assessed using an appropriate filter (550 nm) and quantified by NGL. A double-blind experimental procedure was conducted to remove bias from single-operator measurements. At the end of the experiments, mice were sacrificed and brains were dissected out, snap frozen in liquid nitrogen, and stored at −80 °C until use in Western blot and ELISA assays.

### 2.5. Western Blot Assay

Brain samples were homogenized in 8× volume of radioimmunoprecipitation lysis buffer supplemented with phosphatase and protease inhibitor cocktails (Santa Cruz Biotechnology, Dallas, TX, USA), and protein concentration was measured with the Protein Assay Dye Reagent Concentrate (Bio-Rad Laboratories, Inc., Hercules, CA, USA). For each sample, 30 μg of proteins were run on SDS-PAGE gels (4–20%; Bio-Rad Laboratories, Inc.), and proteins were then transferred onto nitrocellulose membranes (Bio-Rad Laboratories, Inc.). Blots were blocked for 1 h at room temperature with 3% or 5% skimmed milk or 4% bovine serum albumin, depending on the primary antibody subsequently used. Specifications about blocking conditions, codes, and dilutions used for primary antibodies are reported in Table 2. All primary antibodies were left on the respective blots overnight at 4 °C. β-actin was used as an endogenous control. Blots were then incubated for 2 h with appropriate HRP-conjugated secondary antibody (goat anti-rabbit, 170-6515, Bio-Rad Laboratories, Inc.; rabbit anti-mouse, A9044, Sigma-Aldrich, St.Louis, MO, USA; all at 1:5000) and developed using the Clarity Western enhanced chemiluminescence substrate (Bio-Rad Laboratories, Inc.). Images were acquired through the ChemiDoc XRS+ instrument (Bio-Rad Laboratories, Inc.). The optical density (OD) of the target bands was evaluated using the Image Lab 6.0.1 software (Bio-Rad Laboratories, Inc.), and data were normalized to the relative OD of β-actin.

### 2.6. ELISA Assay

Brain samples were homogenized in an 8× volume of homogenization buffer (5 M guanidine-HCl diluted in 50 mM Tris, pH 8.0) containing a protease inhibitor cocktail (Merck, Darmstadt, Germany). The homogenates were sonicated 3 times for 10 s on ice, mixed 6 times for 10 s every 5 min, and centrifugated at 16,000× *g* for 20 min at 4 °C. The supernatants were collected, and the protein concentration was measured with the Protein Assay Dye Reagent Concentrate (Bio-Rad Laboratories, Inc.). Proteins were diluted with standard diluent buffer to an appropriate concentration and used to measure Aβ levels according to the manufacturer’s protocol (mouse Aβ42 ELISA kit KMB3441, Thermo Fisher Scientific, Waltham, MA, USA). The absorbance was determined at 450 nm within 10 min using a microplate reader (FLUOstar Omega version 5.70, BMG Labtech, Ortenberg, Germany). The levels of protein targets in the retinal tissues were calculated using the standard curves and expressed as pg/mg protein.

### 2.7. Novel Object Recognition Test

The novel object recognition (NOR) test was used to assess short- and long-term memory in 5xFAD mice at 2, 4 and 6 months of age, receiving the standard or the ACE oil-enriched diet. This test is based on the innate tendency of rodents to explore novel objects and follows a previously described protocol [50]. Briefly, both WT and 5xFAD mice were used to the arena for 10 min in the absence of objects. In the training phase, two identical objects were presented, and animals were allowed to explore for 5 min. After a 3 h retention interval, one object was replaced with a novel one, and exploration behavior was recorded for 5 min. The next day, 24 h after training, the new object used in the 3 h test was replaced with another new one, and the exploration behavior was recorded again. Video recordings were analyzed using ToxTrac v2.98 (SourceForge, San Diego, CA, USA) to extract mobility parameters and trajectories. Specifically, total distance traveled and mobility rate were quantified during the NOR test sessions at 3 h and 24 h in 5xFAD mice receiving the standard or the ACE oil-enriched diet to exclude potential effects of altered locomotion or exploratory behavior on recognition performance. Trajectories and exploration data were further processed with ImageJ-win32 [51]. To evaluate memory performance, the discrimination index (DI) and the recognition index (RI) were calculated as follows: DI = (Tnovel − Tfamiliar)/(Tnovel + Tfamiliar) and RI = Tnovel/(Tnovel + Tfamiliar), where Tnovel was the exploration time spent near the novel object and Tfamiliar was the exploration time spent near the known object.

### 2.8. Measurement of Body Weight, Water and Food Intake, and Blood Glucose

The body weight was monitored monthly in mice performing the NOR test by placing the animals on a common laboratory balance. Water consumption was monitored weekly by measuring the given and remaining volumes in the water bottles of each cage, and food intake was measured daily by weighing the given and remaining food in each cage. In each case, the result was divided by the number of mice in the cage to obtain the mean consumption of water or food. Glycemia was measured monthly using a OneTouch Ultra glucometer (LifeScan Inc., Milpitas, CA, USA).

### 2.9. Body Fat Mass Measurement, Serum Analysis, and Hepatic Evaluation

WT and 5xFAD mice at 2, 4, and 6 months of age, receiving the standard or the ACE oil-enriched diet, were anesthetized by an intraperitoneal injection of avertin (1.25% solution, 0.02 mL/g body weight). Subsequently, the blood was collected and centrifuged at 16,000× *g* for 5 min at 4 °C. The serum was collected and promptly stored at −80 °C until use. Furthermore, liver tissue was collected in compliance with standard laboratory operating procedures (SOPs), weighed, and snap-frozen at −80 °C for subsequent lipid extraction and quantification or preserved at 4 °C in a fixative solution composed of 70% alcohol (comprising ~60% ethanol and ~40% isopropanol) and 30% distilled water for histopathological examination [52,53].

The amount of body fat mass was quantified by dissecting and weighing the adipose tissue from each animal.

The serum was used to detect total cholesterol, low-density lipoprotein (LDL), high-density lipoprotein (HDL), alkaline phosphatase (ALP), gamma-glutamyltransferase (GGT), and glutamate-pyruvate aminotransferase (GPT). In particular, the LDL, HDL, and total cholesterol serum levels were evaluated using direct colorimetric methods (ASKIT2104, ASKIT1904, ASKIT0802, Assel, Guidonia, Rome), while the serum levels of ALP, GGT, and GPT were quantified using kinetic methods (ASKIT1203, ASKIT1402, ASKIT1702, Assel, Guidonia, Rome) according to the manufacturer’s protocol.

Liver lipid content was quantified using the gravimetric method originally developed by Folch et al. [54], with minor modifications. Mouse liver tissue was homogenized after being mixed with equal volumes of water and methanol. The homogenate underwent three sequential extractions with chloroform, followed by two washes with 1 M KCl and water. The chloroform solution was then completely evaporated and subjected to extended drying until a constant weight was achieved. The lipid content was subsequently quantified and expressed as milligrams of lipids per gram of tissue (mg/g tissue).

To perform histopathological examinations, fixed livers were trimmed according to SOP guidelines, processed, and embedded in paraffin blocks. Sections of 5 µm thickness were cut and routinely stained with hematoxylin and eosin. Histological assessments were conducted under light microscopy in a blinded fashion, with the examiner unaware of treatment allocations [53].

### 2.10. Statistical Analysis

All data were analyzed by the Shapiro–Wilk test to certify normal distribution. Data are expressed as the mean ± standard deviation. Statistical significance was evaluated using two-way ANOVA followed by Tukey’s post hoc multiple comparison test. GraphPad 9.0 software (Prism, San Diego, CA, USA) was used to analyze the data. *p* < 0.05 values were considered statistically significant. To determine the sample size, an a priori power analysis was conducted using the software G*Power 3.0.10 to determine the minimum number of animals necessary to obtain a statistical power of at least 0.80, with α = 0.05, in the presence of a large effect size as expected in these studies. After the data were collected, a post hoc power analysis was conducted in order to confirm that reliable statistical power was obtained in the experiments.

## 3. Results

### 3.1. The ACE Oil-Enriched Diet Prevents Pial Cerebral Microcirculation Alterations

**Figure 1:**
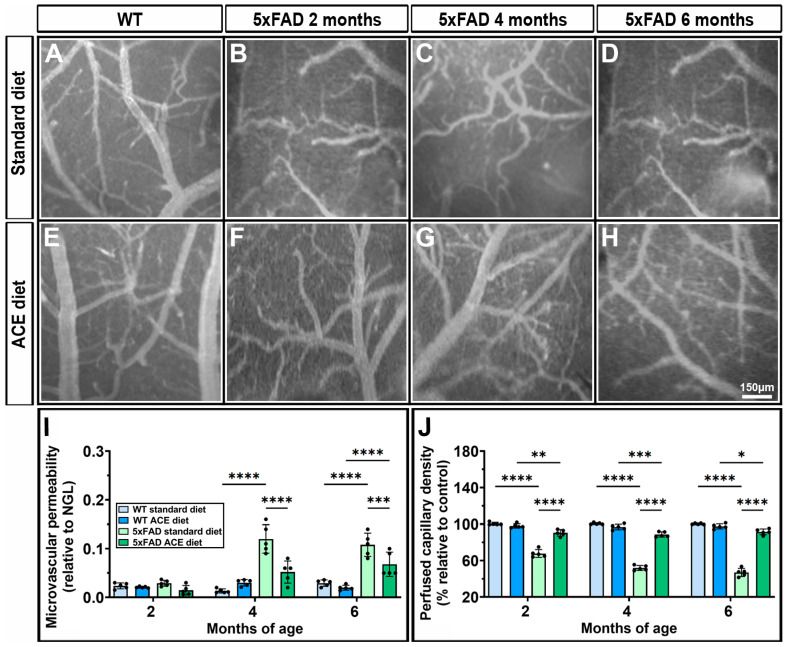
Effects of the Acebuche (ACE) oil-enriched diet on pial microcirculation. (**A**–**H**) Representative images of pial microvasculature in WT (**A**,**E**) and 5xFAD (**B**–**D**,**F**–**H**) mice receiving either the standard (**A**–**D**) or the ACE oil-enriched diet (**E**–**H**). WT mice displayed similar patterns across all ages; therefore, only one representative image is shown for each dietary condition. (**I**,**J**) Quantification of microvascular permeability (**I**) and perfused capillary density (**J**). Data are shown as scatter plots with bars and error bars (mean ± standard deviation (SD)). Statistical significance was evaluated through two-way ANOVA followed by Tukey’s post hoc multiple comparison test (*n* = 5 for each experimental group). * *p* < 0.05, ** *p* < 0.01, *** *p* < 0.001 and **** *p* < 0.0001.

Since cerebral microvasculature is affected in AD patients [7,10,11,12] and EVOO has been reported to positively impact the cerebral microvasculature in subjects with mild cognitive impairment [28], we evaluated the effect of the ACE oil-enriched diet on pial microcirculation. As shown in Figure 1A–D, 5xFAD mice receiving the standard diet displayed far-reaching changes in arteriolar vessel morphology starting at 2 months, as compared to WT.

Using the Straler’s method, three orders of arterioles were identified in all WT mice, while only two orders were present in 5xFAD mice (Table S1). The order 2 vessels in 5xFAD mice were significantly longer than the order 2 vessels in WT mice, a finding attributable to pronounced tortuosity. These characteristics were associated with recurrent arteriovenous anastomoses (Supplementary Videos S1 and S2). The ACE oil-enriched diet significantly reduced the alterations in the pial cerebral microcirculation in 5xFAD mice (Figure 1E–H). In particular, the arteriolar networks were composed of three orders of vessels with a linear shape, similar to WT mice. The microvascular permeability, which increased from 4 months in 5xFAD mice receiving the standard diet, was significantly decreased by the ACE oil-enriched diet (Figure 1I). Moreover, the perfused capillary density, which was progressively reduced from 2 months in 5xFAD mice receiving the standard diet and reached a reduction of about 50% at 6 months, was almost completely restored by the ACE oil-enriched diet (Figure 1J).

### 3.2. The ACE Oil-Enriched Diet Reduces Cerebrovascular Aβ Accumulation

**Figure 2:**
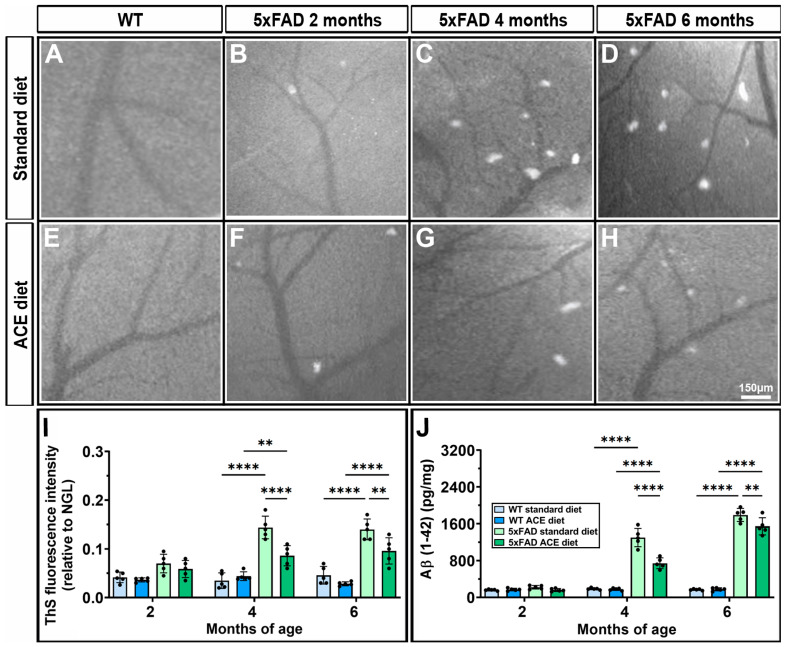
Effects of the ACE oil-enriched diet on cerebrovascular Aβ accumulation. Representative images of thioflavin S (ThS) fluorescent intensity in WT and 5xFAD mice receiving the standard (**A**–**D**) or the ACE oil-enriched diet (**E**–**H**). WT mice displayed similar patterns across all ages; therefore, only one representative image is shown for each dietary condition. (**I**) Quantification of ThS fluorescent intensity. (**J**) Levels of beta-amyloid (Aβ) measured by ELISA in brain homogenates from WT and 5xFAD mice receiving the standard or the ACE oil-enriched diet. Data are shown as scatter plots with bars and error bars (mean ± SD). Statistical significance was evaluated by two-way ANOVA followed by Tukey’s post hoc multiple comparison test (*n* = 5 for each experimental group). ** *p* < 0.01, **** *p* < 0.0001.

ThS is a fluorescent dye commonly used to detect amyloid deposits, typically associated with neurodegenerative diseases such as AD. As shown in Figure 2, the ThS fluorescence intensity was evaluated in WT and 5xFAD mice receiving either the standard diet (Figure 2A–D) or the ACE oil-enriched diet (Figure 2E–H). The data revealed a marked increase in ThS fluorescence intensity (Figure 2I), indicating the presence of widespread Aβ deposits in 5xFAD mice at 4 months (Figure 2C) and 6 months of age (Figure 2D). To corroborate these observations, we quantified brain Aβ levels in WT and 5xFAD mice under both dietary conditions. As illustrated in Figure 2J, 5xFAD mice exhibited a progressive accumulation of Aβ starting at 4 months of age, while dietary supplementation with ACE oil significantly lowered ThS fluorescence intensity and brain Aβ levels in these mice, suggesting a potential protective effect of ACE oil against amyloid pathology.

### 3.3. The ACE Oil-Enriched Diet Prevents the Downregulation of Aβ-Degrading Enzymes

**Figure 3:**
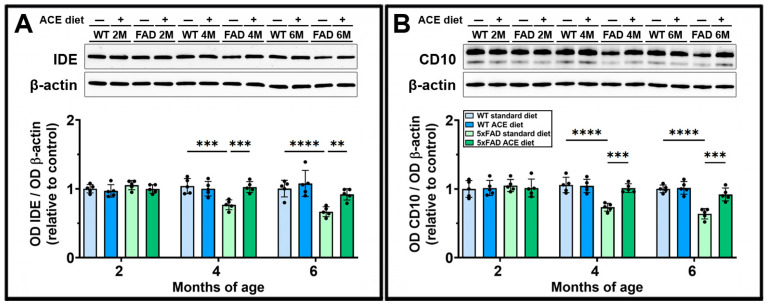
Effects of the ACE oil-enriched diet on levels of Aβ-degrading enzymes. (**A**,**B**) Representative Western blots showing immunoreactive bands and relative densitometric analysis of insulin-degrading enzyme (IDE) (**A**) and cluster of differentiation 10 (CD10) (**B**) levels in brain homogenates from WT and 5xFAD (FAD) mice receiving the standard or the ACE oil-enriched diet. Levels of IDE and CD10 were normalized to β-actin as well as to the control (WT receiving the standard diet at 2 months of age). Data are shown as scatter plots with bars and error bars (mean ± SD). Statistical significance was evaluated through two-way ANOVA followed by Tukey’s post hoc multiple comparison test (*n* = 5 for each experimental group). ** *p* < 0.01, *** *p* < 0.001, and **** *p* < 0.0001.

Since brain Aβ accumulation may result not only from increased production of the peptide but also from its impaired clearance, we evaluated the expression of two key Aβ-degrading enzymes in the brains of both WT and 5xFAD mice receiving either the standard or the ACE oil-enriched diet. As shown in Figure 3, the levels of both insulin-degrading enzyme (IDE) and cluster of differentiation 10 (CD10) were lower in 5xFAD than in WT mice at 4 and 6 months. In 5xFAD mice, the ACE oil-enriched diet prevented the reduction in IDE and CD10 levels.

### 3.4. The ACE Oil-Enriched Diet Prevents Hypoxia and Maintains BBB Integrity

**Figure 4:**
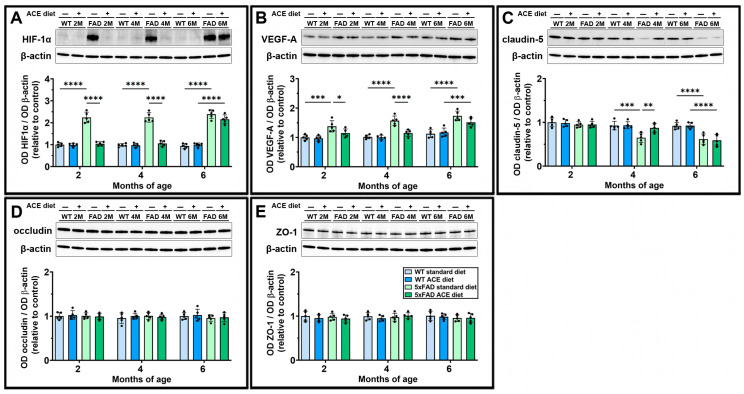
Effects of the ACE oil-enriched diet on levels of hypoxia and blood–brain barrier markers. (**A**–**E**) Representative Western blots showing immunoreactive bands and relative densitometric analysis of hypoxia-inducible factor 1α (HIF-1α) (**A**), vascular endothelial growth factor type A (VEGF-A) (**B**), claudin-5 (**C**), occludin (**D**), and zonula occludens (ZO)-1 (**E**) levels in brain homogenates from WT and 5xFAD (FAD) mice receiving the standard or the ACE oil-enriched diet. Levels of HIF-1α, VEGF-A, claudin-5, occludin, and ZO-1 were normalized to β-actin as well as to the control (WT receiving the standard diet at 2 months of age). Data are shown as scatter plots with bars and error bars (mean ± SD). Statistical significance was evaluated through two-way ANOVA followed by Tukey’s post hoc multiple comparison test (*n* = 5 for each experimental group). * *p* < 0.05, ** *p* < 0.01, *** *p* < 0.001 and **** *p* < 0.0001.

The presence of hypoxic conditions, likely resulting from vascular alterations, and the progressive breakdown of BBB integrity were assessed by Western blot (Figure 4). A marked increase in hypoxia-inducible factor 1α (HIF-1α) (Figure 4A) and vascular endothelial growth factor type A (VEGF-A) (Figure 4B) levels was found in 5xFAD mice starting from 2 months of age. In 5xFAD mice, the ACE oil-enriched diet restored both HIF-1α and VEGF-A levels at 2 and 4 months but not at 6 months. Moreover, from 4 months onward, 5xFAD mice exhibited a significant reduction in cerebral claudin-5 expression (Figure 4C), whose levels were restored by the ACE oil-enriched diet at 4 months but not at 6 months. The levels of other BBB markers, such as occludin (Figure 4D) and zonula occludens-1 (ZO-1) (Figure 4E), were not affected in 5xFAD mice.

### 3.5. The ACE Oil-Enriched Diet Reduces Gliosis

**Figure 5:**
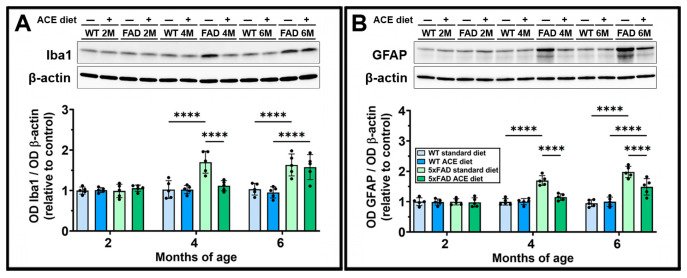
Effects of the ACE oil-enriched diet on levels of micro- and macrogliosis markers. (**A**,**B**) Representative Western blots showing immunoreactive bands and relative densitometric analysis of ionized calcium-binding adaptor molecule 1 (Iba1) (**A**) and glial fibrillary acidic protein (GFAP) (**B**) levels in brain homogenates from WT and 5xFAD (FAD) mice receiving the standard or the ACE oil-enriched diet. Levels of Iba1 and GFAP were normalized to β-actin as well as to the control (WT receiving the standard diet at 2 months of age). Data are shown as scatter plots with bars and error bars (mean ± SD). Statistical significance was evaluated through two-way ANOVA followed by Tukey’s post hoc multiple comparison test (*n* = 5 for each experimental group). **** *p* < 0.0001.

To investigate whether Aβ accumulation and BBB alterations might trigger reactive gliosis and if this process might be counteracted by ACE oil, we evaluated the expression of the acknowledged markers of micro- and macrogliosis ionized calcium-binding adaptor molecule 1 (Iba1) and glial fibrillary acidic protein (GFAP), respectively. As shown in Figure 5, both Iba1 (Figure 5A) and GFAP (Figure 5B) were upregulated in the brain of 5xFAD mice starting from 4 months. The ACE oil-enriched diet significantly attenuated both micro- and macrogliosis in 4-month-old mice, with a loss of effectiveness on microgliosis at 6 months of age.

### 3.6. The ACE Oil-Enriched Diet Delays the Onset of Neuroinflammatory Processes

**Figure 6:**
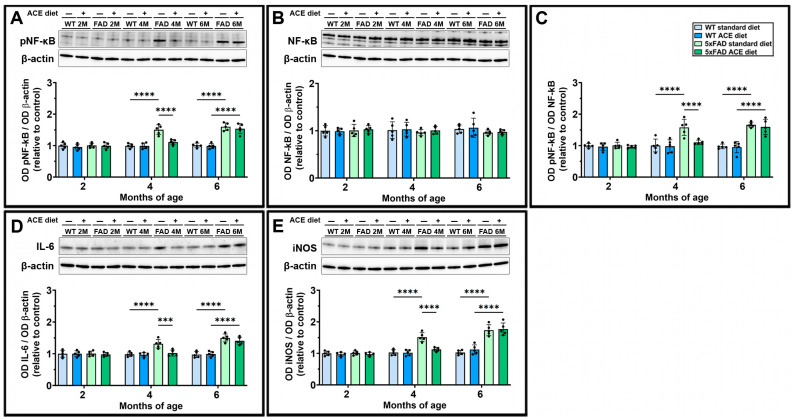
Effects of the ACE oil-enriched diet on levels of neuroinflammatory markers. (**A**,**B**) Representative Western blots showing immunoreactive bands and relative densitometric analysis of the phosphorylated form of nuclear factor kappa-light-chain-enhancer of activated B cells (pNF-kB) (**A**) and its total form (NF-kB) (**B**) in brain homogenates from WT and 5xFAD (FAD) mice receiving the standard or the ACE oil-enriched diet. (**C**) Ratio between the levels of pNF-kB and NF-kB. (**D**,**E**) Representative Western blots showing immunoreactive bands and relative densitometric analysis of interleukin 6 (IL-6) (**D**) and the inducible form of nitric oxide synthase (iNOS) (**E**) in the same groups as in (**A**,**B**). Levels of pNF-kB, NF-kB, IL-6, and iNOS were normalized to β-actin as well as to the control (WT receiving the standard diet at 2 months of age). Data are shown as scatter plots with bars and error bars (mean ± SD). Statistical significance was evaluated through two-way ANOVA followed by Tukey’s post hoc multiple comparison test (*n* = 5 for each experimental group). *** *p* < 0.001 and **** *p* < 0.0001.

Cerebral gliosis is accompanied by the activation of neuroinflammatory processes. As shown in Figure 6, starting from 4 months, 5xFAD mice exhibited clear signs of neuroinflammation. This was evidenced by an increased ratio between the phosphorylated form of nuclear factor kappa-light-chain-enhancer of activated B cells (pNF-kB) and its total form (Figure 6A–C), elevated levels of the pro-inflammatory cytokine interleukin 6 (IL-6) (Figure 6D) and upregulation of a key player in the brain inflammatory response, the inducible form of nitric oxide synthase (iNOS) (Figure 6E). In 5xFAD mice, the ACE oil-enriched diet significantly attenuated the neuroinflammatory markers at 4 months, whereas this effect was no longer observed at 6 months.

### 3.7. The ACE Oil-Enriched Diet Ameliorates Cognitive Deficits

**Figure 7:**
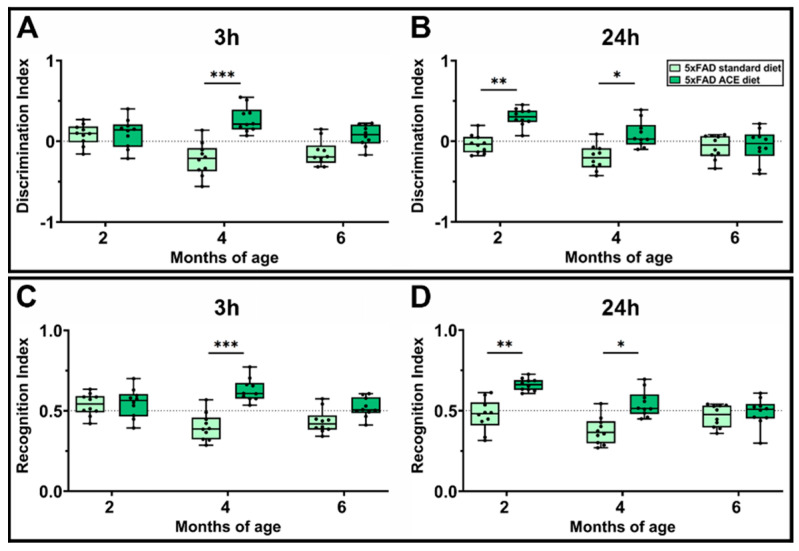
Effects of the ACE oil-enriched diet on cognitive functions. Cognitive functions in 5xFAD mice receiving the standard or the ACE oil-enriched diet were assessed by the novel object recognition test. (**A**–**D**) Discrimination index (**A**,**B**) and recognition index (**C**,**D**) evaluated at 3 h (**A**,**C**) and 24 h (**B**,**D**) after training. Data are shown as box plots with minimum to maximum whiskers. Statistical significance was evaluated through two-way ANOVA followed by Tukey’s post hoc multiple comparison test (*n* = 10 for each experimental group). * *p* < 0.05, ** *p* < 0.01, *** *p* < 0.001.

The cognitive decline in 5xFAD mice typically begins at 4 months of age, marking the onset of a prodromal phase that progresses to a symptomatic phase by approximately 6 months [55]. To investigate the potential neuroprotective effects of the ACE oil-enriched diet on cognitive function, 5xFAD mice receiving the standard or the ACE oil-enriched diet were subjected to the NOR test by evaluating both short- (3 h) and long-term (24 h) memory. As shown in Figure 7, the two-key metrics, DI and RI, were ameliorated by the ACE oil, with an effectiveness that was lost at 6 months. In particular, when tested at 3 h, DI and RI were increased at 4 months, while when tested at 24 h, DI and RI resulted in increased values at both 2 and 4 months. The ameliorative effects of the ACE oil-enriched diet were not associated with changes in locomotor activity, as total distance traveled and mobility rate during the NOR test were comparable between mice receiving the standard diet and mice receiving the ACE oil-enriched diet at all ages and testing times (Figure S1).

### 3.8. Effects of the ACE Oil-Enriched Diet on Metabolic Parameters

**Figure 8:**
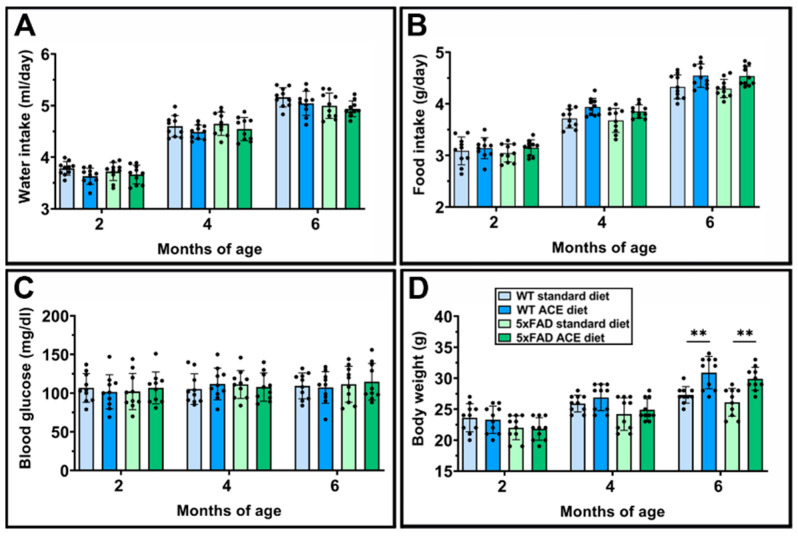
Effects of the ACE oil-enriched diet on metabolic parameters. Water intake (**A**), food intake (**B**), blood glucose (**C**), and body weight (**D**) in WT and in 5xFAD mice receiving the standard or the ACE oil-enriched diet. Data are shown as scatter plots with bars and error bars (mean ± SD). Statistical significance was evaluated through two-way ANOVA followed by Tukey’s post hoc multiple comparison test (*n* = 10 for each experimental group). ** *p* < 0.01.

We monitored the effects of the ACE oil-enriched diet on metabolic parameters. As shown in Figure 8, no differences were observed in water consumption (Figure 8A), food intake (Figure 8B), and blood glucose levels (Figure 8C) among the experimental groups over time. Conversely, body weight was significantly increased in both WT and 5xFAD mice receiving the ACE oil-enriched diet at 6 months (Figure 8D).

**Figure 9:**
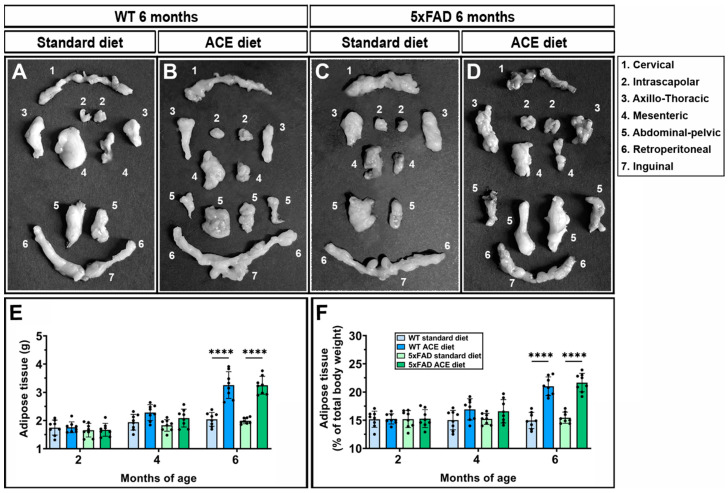
Effects of the ACE oil-enriched diet on adiposity. (**A**–**D**) Representative images of adipose tissue harvested from 6-month-old WT and 5xFAD mice receiving the standard or the ACE oil-enriched diet. (**E**,**F**) Quantification of adipose tissue expressed as absolute weight (**E**) or as a percentage of body weight (**F**). Data are shown as scatter plots with bars and error bars (mean ± SD). Statistical significance was evaluated through two-way ANOVA followed by Tukey’s post hoc multiple comparison test (*n* = 8 for each experimental group). **** *p* < 0.0001.

At 2 and 4 months of age, no difference in adipose tissue content was observed in WT and 5xFAD mice receiving the standard or the ACE oil-enriched diet. In contrast, at 6 months of age, both WT and 5xFAD mice receiving the ACE oil-enriched diet exhibited a significantly increased amount of adipose tissue compared to their respective counterparts receiving the standard diet (Figure 9).

**Figure 10:**
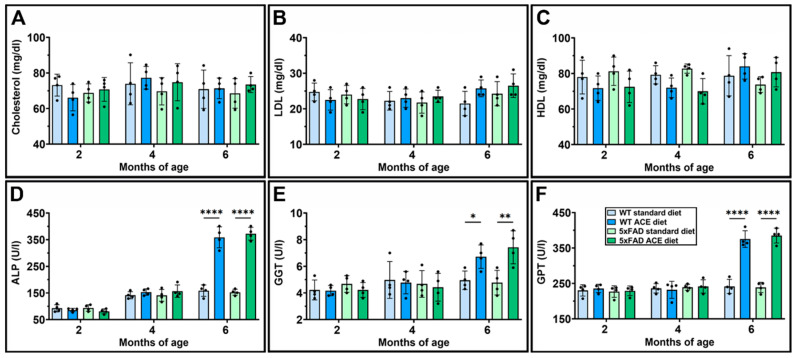
Effects of the ACE oil-enriched diet on serum levels of cholesterol and hepatic enzymes. (**A**–**F**) Serum levels of total cholesterol (**A**), low-density lipoprotein (LDL) (**B**), high-density lipoprotein (HDL) (**C**), alkaline phosphatase (ALP) (**D**), gamma-glutamyltransferase (GGT) (**E**), and glutamate-pyruvate-aminotransferase (**F**). Data are shown as scatter plots with bars and error bars (mean ± SD). Statistical significance was evaluated through two-way ANOVA followed by Tukey’s post hoc multiple comparison test (*n* = 4 for each experimental group). * *p* < 0.05, ** *p* < 0.01 and **** *p* < 0.0001.

Serum biochemical analyses were conducted to evaluate cholesterol levels (Figure 10A–C), as well as the levels of some key hepatic enzymes (Figure 10D–F). No significant differences were observed in total cholesterol (Figure 10A), LDL (Figure 10B), or HDL (Figure 10C) levels among the experimental groups. Conversely, serum levels of hepatic enzymes, including ALP (Figure 10D), GGT (Figure 10E), and GPT (Figure 10F), were considerably increased in both 6-month-old WT and 5xFAD mice receiving the ACE oil-enriched diet.

### 3.9. Quantitation of Liver Lipids and Histopathological Analysis

**Figure 11:**
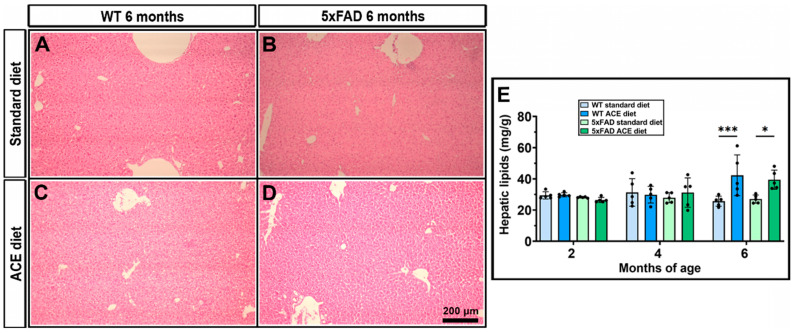
Effects of the ACE oil-enriched diet on hepatic lipid content. (**A**–**D**) Hematoxylin and eosin staining of liver tissue from 6-month-old WT and 5xFAD mice receiving the standard or the ACE oil-enriched diet. (**E**) Total hepatic lipid content. Data are shown as scatter plots with bars and error bars (mean ± SD). Statistical significance was evaluated through two-way ANOVA followed by Tukey’s post hoc multiple comparison test (*n* = 5 for each experimental group). * *p* < 0.05 and *** *p* < 0.001.

Through histopathological analysis and gravimetric lipid quantification, we aimed to determine whether oil supplementation could induce hepatic lipid accumulation. These assessments were performed to evaluate potential morphological and quantitative alterations in liver lipid content at the three different experimental time points, thereby elucidating the temporal effects of oil intake on hepatic lipid metabolism. Although no differences in hepatic lipid content were apparently observed in 2- and 4-month-old WT and 5xFAD receiving the standard or the ACE oil-enriched diet, histological analysis revealed marked lipid accumulation in the liver of 6-month-old animals receiving the ACE oil-enriched diet (Figure 11A–D). As shown in Figure 11A,B, liver sections from animals receiving the standard diet displayed a healthy architecture characterized by well-defined nuclei and well-preserved cytoplasm, without any evidence of steatosis, while liver sections from mice receiving the ACE oil-enriched diet showed morphological alterations typical of hepatic steatosis (Figure 11C,D). This qualitative data was supported by the gravimetric analysis (Figure 11E), which confirmed the presence of a higher amount of hepatic lipids in both 6-month-old WT and 5xFAD mice receiving the ACE oil-enriched diet.

## 4. Discussion

This work provides a comprehensive overview of the pial microcirculation profile and the main brain molecular features of the 5xFAD murine model of AD, as well as new evidence supporting the protective effect of ACE oil against AD-related neurovascular and molecular changes.

Under physiological conditions, the proper functioning of the cerebral vasculature plays a pivotal role in maintaining CNS homeostasis [56,57]. A growing body of evidence suggests that cerebrovascular dysfunction is a key factor in the development of various neurodegenerative disorders, including AD [58,59]. In line with this, our fluorescence microscopy analyses revealed clear morpho-functional impairments in the cerebral pial microcirculation of 5xFAD mice as early as 2 months of age, consistent with current literature [40,42,60,61,62]. In addition to the observed cerebrovascular changes, 5xFAD mice show lower levels of claudin-5, one of the most abundantly expressed tight junction proteins in the CNS, whose deficiency has been demonstrated to contribute to neurodegenerative disease progression as in AD [63]. Besides that, increased brain levels of HIF-1α and VEGF-A can be observed starting from 2 months, suggesting the presence of hypoxic conditions, a well-recognized feature of AD pathology typically resulting from vascular dysfunction [64,65,66]. The long-lasting activation of these factors may lead to the development of inflammatory processes and neurovascular alterations [67,68]. HIF-1 has also been reported to downregulate Aβ-degrading enzymes and concurrently to stimulate β-secretase and γ-secretase enzymatic complex activity, thereby exacerbating Aβ accumulation and accelerating AD progression [67,69,70]. These alterations coincide with increased cerebral Aβ levels that can be observed later than the onset of vascular damage, aligning with the widely supported “two-hit vascular hypothesis”, which proposes a two-step positive feedback loop in which an initial vascular insult compromises BBB integrity and Aβ clearance mechanisms, thereby creating a permissive environment for subsequent Aβ accumulation and neurodegeneration [56,71,72]. In addition, Aβ accumulation in the perivascular spaces can result in a gradual narrowing of the vascular lumen, ultimately leading to hypoxia and activation of HIF-1α, which further exacerbates the neuroinflammatory response and contributes to subsequent vascular damage [73,74]. A fundamental role in the development of AD-related neuroinflammatory processes is played by the transcription factor NF-κB, which can be induced by Aβ to orchestrate the expression of a wide range of pro-inflammatory mediators [75,76]. Moreover, NF-κB can transcriptionally regulate β-secretase expression, thus affecting the amyloidogenic pathway and cerebral Aβ accumulation [77,78,79]. The progressive formation of Aβ deposits in the brain may also promote the recruitment and consequent activation of astrocytes and microglia [80]. In the early stages of AD, glial reactivity plays a protective role by enhancing receptor-mediated phagocytosis of Aβ; however, chronic activation leads to an impaired phagocytic capacity, accompanied by a shift toward a pro-inflammatory phenotype, contributing to neuroinflammation, synaptic dysfunction, and cognitive deficits [80,81,82]. As expected, in 5xFAD mice the concomitant presence of an altered microvascular network and marked molecular alterations leads to the progressive memory loss starting from 4 months, confirming the findings of previous studies [83,84,85]. Overall, results from our study confirm that 5xFAD mice represent an optimal model to evaluate whether an ACE oil-enriched diet may have a positive influence on the development or progression of neurodegenerative diseases.

Besides drug therapies and genetic approaches [76,86,87,88], a growing body of evidence suggests that regular consumption of foods rich in anti-inflammatory compounds, antioxidants, B-group vitamins, and polyunsaturated fatty acids, such as EVOO, may exert beneficial effects in slowing down AD progression [26,28,89]. The administration of an ACE oil-enriched diet, well-known for its neuroprotective properties [35,36], effectively prevents the onset of cerebral microcirculatory abnormalities in 5xFAD mice as early as 2 months of age, by preserving microvascular architecture and function. ACE oil also significantly alleviates the hypoxic condition observed in 5xFAD mice, preserving BBB integrity and preventing the development of inflammatory processes, ultimately resulting in a marked reduction in brain Aβ and a significant improvement in cognitive function. Overall, these findings suggest that the components in which the ACE oil is particularly rich exert broad-spectrum effects, supporting previous data demonstrating the higher efficacy of the ACE oil in exerting anti-inflammatory and neuroprotective effects with respect to EVOO [32,33,36]. However, despite ACE oil administration conferring a marked protection against early neurovascular and molecular alterations, its efficacy is reduced at 6 months, with ACE oil still capable of preventing some AD-related brain alterations but not others. The reduced effectiveness of the ACE oil coincides with increased body weight, total adipose tissue, and hepatic lipid content, along with higher serum levels of some liver functionality-related enzymes, suggesting that the development of a systemic dysmetabolic state characterized by hepatic steatosis may have overridden the initial protective effects of ACE oil. In this respect, metabolic imbalance in the liver has been associated with AD [90], indicating that alterations in the liver-brain crosstalk may play a role in AD pathogenesis. In addition, mice with hepatic steatosis show an increased accumulation of APP in the liver that decreases the scavenging action of the liver towards Aβ, potentially enhancing Aβ accumulation in the periphery, including the brain, thus promoting AD [91]. Moreover, in a rat model of chronic liver disease, liver dysfunction has been demonstrated to associate with central and systemic AD-related markers, including Aβ and hyperphosphorylated Tau protein [92]. In general, data from preclinical models point on liver dysfunction as an early event in AD pathogenesis, with altered Aβ clearance and inflammation [93]. The association between markers of liver function and cognitive performance has also been observed in AD patients, although whether this association is causative of the disease or it has a secondary role remains to be established [94]. However, the hepatic damage may not be the only explanation for the reduced effectiveness of the diet at 6 months. Indeed, this may also be related to the fact that the 5xFAD mouse, carrying 5 AD-linked mutations, is a model with a rapid onset of the AD phenotype, in particular when compared with other mouse models of AD [95]. Therefore, it is plausible that the anti-inflammatory components of the ACE oil may face such a high burden of metabolic stress that they lose efficacy after months of administration. Although this hypothesis needs to be verified, for instance using a model with a milder AD-related phenotype, the present findings represent proof of concept that the introduction of an EVOO rich in anti-inflammatory components into the diet may be protective against AD-related damage. On the other hand, the metabolic imbalance after months of the ACE oil-enriched diet was expected since the lipid content of the standard diet is roughly 6% whereas the content in the ACE oil-enriched diet is about 17%. In a translational perspective, therefore, to maintain the long-term efficacy of the diet, alternative strategies could be based on a reduced dietary concentration of ACE oil that would make long-term dietary treatments feasible.

Finally, we must consider possible limitations of this study, in which data from male and female 5xFAD mice have been mixed together. It is indeed known that female 5xFAD mice express higher levels of APP, Aβ and neuroinflammatory markers than male mice [96], suggesting that our results may have underestimated the effectiveness of the ACE oil-enriched diet on some parameters. On the other hand, a high-fat, high carbohydrate diet induces a release of inflammatory cytokines in both male and female 5xFAD mice, with an increased Aβ production in male but not in female mice [97].

## 5. Conclusions

Cerebrovascular dysfunction is increasingly recognized as an early and central event in AD pathogenesis. Vascular alterations often precede the typical AD hallmarks, such as Aβ accumulation, neuroinflammation, synaptic dysfunction, and cognitive impairment, suggesting a key upstream role in disease progression. In this context, the implementation of effective therapeutic strategies becomes crucial, especially those based on lifestyle interventions. Among these, dietary approaches emerge as a promising avenue for preserving cerebrovascular health and lowering the risk of AD onset. In this study, the ACE oil-enriched diet effectively prevented early neurovascular and molecular alterations in 5xFAD mice, including microvascular dysfunction, hypoxia, tight junction disruption, Aβ accumulation, and neuroinflammation, while also improving cognitive performance. However, its reduced efficacy at later stages suggests that prolonged administration may be hindered and limited by systemic metabolic changes, such as hepatic steatosis. These findings highlight the potential of early dietary interventions in AD prevention, while also underscoring the need for optimized long-term strategies that consider both disease progression and metabolic balance.

## Figures and Tables

**Table 1:** List of primers used for genotyping experiments.

Primer	Sequence (5′ -> 3′)	Catalog
Mutant Reverse	CGG GCC TCT TCG CTA TTA C	27,367
WT Reverse	TAT ACA ACC TTG GGG GAT GG	37,599
Common Forward	ACC CCC ATG TCA GAG TTC CT	37,598

**Table 2:** List of primary antibodies used for Western Blot experiments.

Antibody	Source	Catalog	Blocking	Dilution
Rabbit polyclonal antibody anti-IDE	Abcam	ab32216	5% skimmed milk	1:1000 in 5% skimmed milk
Rabbit monoclonal antibody anti-CD10	Abcam	ab256494	5% skimmed milk	1:1000 in 5% skimmed milk
Rabbit monoclonal antibody anti-HIF1α	Abcam	ab179483	5% skimmed milk	1:1000 in 5% skimmed milk
Rabbit monoclonal antibody anti-VEGFA	Abcam	ab214424	5% skimmed milk	1:1000 in 5% skimmed milk
Mouse monoclonal antibody anti-claudin 5	Invitrogen	35-2500	5% skimmed milk	1:500 in 5% skimmed milk
Rabbit monoclonal antibody anti-occludin	Abcam	ab216327	5% skimmed milk	1:1000 in 5% skimmed milk
Rabbit polyclonal antibody anti-ZO1	Abcam	ab96587	5% skimmed milk	1:500 in 5% skimmed milk
Rabbit monoclonal antibody anti-Iba1	Abcam	ab178846	5% skimmed milk	1:500 in 3% skimmed milk
Rabbit monoclonal antibody anti-GFAP	Abcam	ab207165	5% skimmed milk	1:5000 in 5% skimmed milk
Rabbit monoclonal antibody anti-pNF-κB (p65)	Abcam	ab76302	5% skimmed milk	1:1000 in 4% BSA
Rabbit polyclonal antibody anti-NF-κB (p65)	Abcam	ab16502	5% skimmed milk	1:1000 in 5% skimmed milk
Mouse monoclonal antibody anti-IL-6	Santa Cruz Biotechnology	sc57315	5% skimmed milk	1:200 in 5% skimmed milk
Rabbit monoclonal antibody anti-iNOS	Abcam	ab178945	5% skimmed milk	1:1000 in 5% skimmed milk
Mouse monoclonal antibody anti-β-actin	Sigma-Aldrich	A2228	5% skimmed milk	1:2500 in 5% skimmed milk

## Data Availability

The original contributions presented in this study are included in the article/Supplementary Materials. Further inquiries can be directed to the corresponding author.

## Supplementary Materials

The following supporting information can be downloaded at: https://www.mdpi.com/article/10.3390/nu18010172/s1, Figure S1: Effects of the ACE oil-enriched diet on locomotor activity; Table S1: Classification of pial arterioles in the different experimental groups according to the Strahler’s scheme.title; Video S1: WT standard diet; Video S2: 5xFAD standard diet.

## Abbreviations

The following abbreviations are used in this manuscript:

AD	Alzheimer’s disease
Aβ	Beta-amyloid
EVOO	Extra virgin olive oil
BBB	Blood–brain barrier
APP	Amyloid precursor protein
ACE	Acebuche
PSEN1	Presenilin 1
WT	Wild-type
FITC	Fluorescein isothiocyanate
NGL	Normalized gray level
ThS	Thioflavin-S
OD	Optical density
NOR	Novel object recognition
DI	Discrimination index
RI	Recognition index
SOPs	Standard laboratory operating procedures
LDL	Low-density lipoprotein
HDL	High-density lipoprotein
ALP	Alkaline phosphatase
GGT	Gamma glutamyltransferase
GPT	Glutamate-pyruvate aminotransferase
SD	Standard deviation
IDE	Insulin-degrading enzyme
CD10	Cluster of differentiation 10
HIF-1α	Hypoxia-inducible factor 1α
VEGF-A	Vascular endothelial growth factor type A
ZO-1	Zonula occludens-1
Iba1	Ionized calcium-binding adaptor molecule 1
GFAP	Glial fibrillary acidic protein
NF-kB	Nuclear factor kappa-light-chain-enhancer of activated B cells
IL-6	Interleukin 6
iNOS	Inducible form of nitric oxide synthase
