# Supplementary material for: An Acebuche Oil-Enriched Diet Prevents Early-Stage Cerebrovascular Alterations in the 5xFAD Mouse Model of Alzheimer’s Disease
Source: Nutrients. 2026 Jan 5;18(1):172. doi: 10.3390/nu18010172 (PMC12787596; doi:10.3390/nu18010172)
Supplement: Supplementary file 1 [file nutrients-18-00172-s001.zip › Figure S1.pdf]

Figure S1.

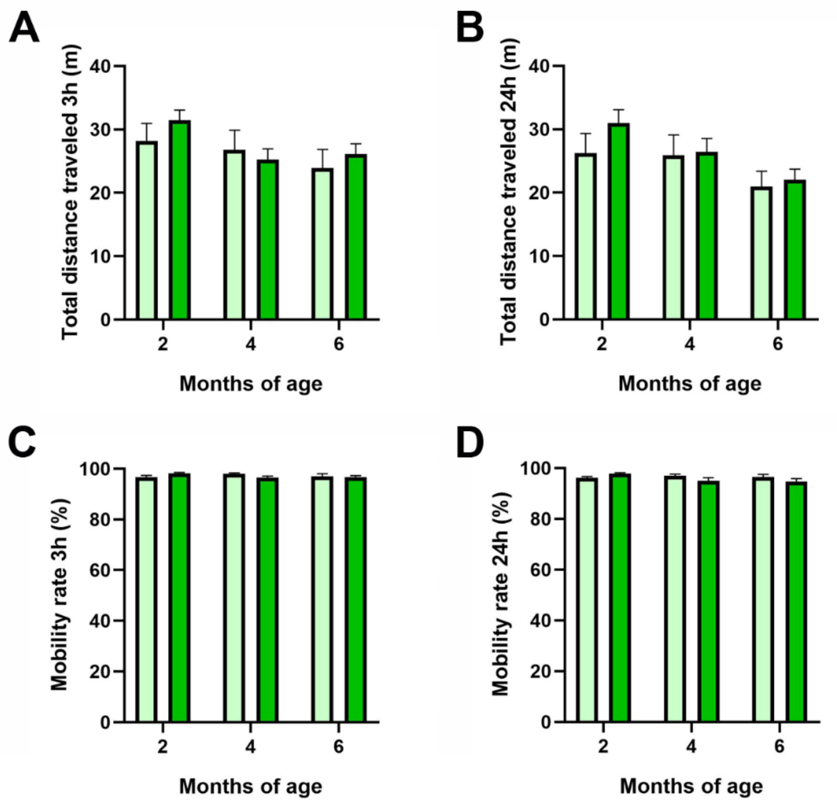

Figure S1. Effects of the ACE oil-enriched diet on locomotor activity. Locomotor activity in 5xFAD mice receiving the standard or the ACE oil-enriched diet was assessed during the novel object recognition (NOR) test. (**A,B**) Total distance traveled (m) during the 3 h (**A**) or the 24 h (**B**) NOR test. (C,D) Mobility rate (%) during the 3 h (**C**) or the 24 h (**D**) NOR test. Data are shown as mean ± SD. Statistical significance was evaluated through two-way ANOVA followed by Tukey's post-hoc multiple comparison test (n = 10 for each experimental group).
