# Supplementary material for: An Acebuche Oil-Enriched Diet Prevents Early-Stage Cerebrovascular Alterations in the 5xFAD Mouse Model of Alzheimer’s Disease
Source: Nutrients. 2026 Jan 5;18(1):172. doi: 10.3390/nu18010172 (PMC12787596; doi:10.3390/nu18010172)
Supplement: Supplementary file 1 [file nutrients-18-00172-s001.zip › Table S1.pdf]

Table S1. Classification of pial arterioles in the different experimental groups according to the Strahler's scheme.

Experimental Group	Dieta	Order	Diameter (μm)	Length (μm)	Arterioles (n)
2-month-old WT	Standard	3	36.9 ± 2.4	587.0 ± 14.3	2.0 ± 1.0
		2	24.5 ± 1.7	325.7 ± 21.2	5.5 ± 1.0
		1	16.0 ± 0.9	174.5 ± 18.0	7.0 ± 2.0
2-month-old WT	Ace oil	3	36.3 ± 2.2	580.0 ± 15.5	2.0 ± 0.5
		2	25.7 ± 2.3	320.8 ± 20.2	4.0 ± 1.0
		1	17.1 ± 0.9	168.3 ± 17.0	8.0 ± 2.0
2-month-old 5xFAD	Standard	3	None	None	None
		2	26.3 ± 2.1	405.2 ± 16.8***	2.5 ± 0.5*
		1	15.5 ± 0.5	205.8 ± 12.5*	4.5 ± 1.0
2-month-old 5xFAD	Ace oil	3	34.8 ± 2.3	489.1 ± 16.7	1.0 ± 0.5
		2	23.9 ± 1.4	384.4 ± 19.2	4.0 ± 1.5
		1	17.3 ± 0.2	168.7 ± 15.3§§	5.0 ± 1.0
4-month-old WT	Standard	3	36.6 ± 3.2	622.3 ± 12.2	2.0 ± 1.0
		2	25.8 ± 2.6	405.8 ± 17.6	5.0 ± 1.0
		1	16.7 ± 1.3	180.3 ± 16.0	6.0 ± 2.0
4-month-old WT	Ace oil	3	35.9 ± 2.0	657.4 ± 15.3	1.5 ± 0.5
		2	26.7 ± 1.9	397.2 ± 12.9	5.0 ± 1.5

		1	16.2 ± 1.7	195.3 ± 9.6	7.0 ± 1.0
4-month-old 5xFAD	Standard	3	None	None	None
		2	25.1 ± 3.6	478.1 ± 20.4***	2.0 ± 0.5*
		1	17.0 ± 1.4	189.0 ± 15.7	4.0 ± 1.0
4-month-old 5xFAD	Ace oil	3	35.2 ± 3.0	623.7 ± 15.4	1.0 ± 0.5
		2	24.8 ± 2.6	352.3 ± 17.2§§§	4.0 ± 1.5
		1	16.7 ± 1.7	176.5 ± 14.7	6.0 ± 1.0
6-month-old WT	Standard	3	37.1 ± 3.0	635.7 ± 13.1	1.5 ± 1.0
		2	26.4 ± 1.8	373.6 ± 18.7	6.0 ± 0.5
		1	17.1 ± 0.3	202.5 ± 14.4	7.0 ± 1.5
6-month-old WT	Ace oil	3	36.8 ± 2.2	627.5 ± 13.8	2.0 ± 1.0
		2	25.1 ± 1.6	303.0 ± 15.9	6.5 ± 2.0
		1	16.7 ± 0.8	197.5 ± 15.8	8.0 ± 2.0
6-month-old 5xFAD	Standard	3	None	None	None
		2	27.2 ± 2.3	525.6 ± 17.3***	1.5 ± 0.5*
		1	15.9 ± 1.9	195.4 ± 11.1	3.5 ± 1.5
6-month-old 5xFAD	Ace oil	3	36.7 ± 2.5	503.7 ± 15.2	2.5 ± 1.0
		2	24.9 ± 2.7	408.1 ± 21.5§§§	5.0 ± 1.0
		1	15.5 ± 0.8	188.4 ± 13.8	7.0 ± 2.0

Arterioles (n): total number of arterioles identified within each vessel order. Statistical significance was evaluated through unpaired Student's *t* test (n = 5 for each experimental group). * $p < 0.05$ and ** $p < 0.001$ vs. WT mice with standard diet of

the age-matched group; $^{§§}p < 0.01$ and $^{§§§}p < 0.001$ vs. 5xFAD mice with Ace oil diet of the age-matched group treated with ACE-OIL.
